# A novel extended form of alpha-synuclein 3′UTR in the human brain

**DOI:** 10.1186/s13041-018-0371-x

**Published:** 2018-05-25

**Authors:** Goun Je, Subhrangshu Guhathakurta, Seung Pil Yun, Han Seok Ko, Yoon-Seong Kim

**Affiliations:** 1 Burnett School of Biomedical Sciences, College of Medicine, University of Central Florida, Orlando, FL USA; 2 Neuroregeneration and Stem Cell Programs, Institute for Cell Engineering, The Johns Hopkins University School of Medicine, Baltimore, MD USA; 3 Department of Neurology, The Johns Hopkins University School of Medicine, Baltimore, MD USA; 4 Adrienne Helis Malvin Medical Research Foundation, New Orleans, LA USA; 5 Diana Helis Henry Medical Research Foundation, New Orleans, LA USA; 6 College of Medicine, Kyung-Hee University, Seoul, South Korea

**Keywords:** Parkinson’s disease, Alpha-synuclein, 3′ untranslated region (3′UTR), mRNA

## Abstract

Alpha-synuclein (α-SYN) is one of the key contributors in Parkinson’s disease (PD) pathogenesis. Despite the fact that increased α-SYN levels are considered one of the key contributors in developing PD, the molecular mechanisms underlying the regulation of α-SYN still needs to be elucidated. Since the 3′ untranslated regions (3′UTRs) of messenger RNAs (mRNAs) have important roles in translation, localization, and stability of mRNAs through RNA binding proteins (RBPs) and microRNAs (miRNAs), it is important to identify the exact length of 3′UTRs of transcripts in order to understand the precise regulation of gene expression. Currently annotated human α-SYN mRNA has a relatively long 3′UTR (2529 nucleotides [nt]) with several isoforms. RNA-sequencing and epigenomics data have suggested, however, the possible existence of even longer transcripts which extend beyond the annotated α-SYN 3′UTR sequence. Here, we have discovered the novel extended form of α-SYN 3′UTR (3775 nt) in the *substantia nigra* of human postmortem brain samples, induced pluripotent stem cell (iPSC)-derived dopaminergic neurons, and other human neuronal cell lines. Interestingly, the longer variant reduced α-SYN translation. The extended α-SYN 3′UTR was significantly lower in iPSC-derived dopaminergic neurons from sporadic PD patients than controls. On the other hand, α-SYN protein levels were much higher in PD cases, showing the strong negative correlation with the extended 3′UTR. These suggest that dysregulation of the extended α-SYN 3′UTR might contribute to the pathogenesis of PD.

**Electronic supplementary material:**

The online version of this article (10.1186/s13041-018-0371-x) contains supplementary material, which is available to authorized users.

## Introduction

α-synuclein (α-SYN) is the major component of Lewy bodies (LBs) and Lewy neurites (LNs), the pathological hallmarks of Parkinson’s disease (PD) [1]. Mutations and multiplication of *SNCA* gene coding for α-SYN protein have been strongly implicated in familial forms of PD [2–4]. Furthermore, in sporadic PD, the significant increase in α-SYN expression has been reported [5, 6]. However, the molecular mechanisms underlying the regulation of α-SYN expression that leads to the pathogenesis of PD remain unclear.

The 3′ untranslated regions (3′UTRs) of messenger RNAs (mRNAs) play important roles in translation, localization, and stability of mRNAs through providing binding sites for RNA binding proteins (RBPs) and microRNAs (miRNAs) [7]. Different lengths of the 3′UTRs are generated through alternative polyadenylation, and 3′UTR isoforms vary across tissue types [8–11]. It is noteworthy that neurons usually have transcripts with much longer 3′UTRs, suggesting a more complicated regulation of protein expression in this highly polarized cell [12–14]. Therefore, it is important to identify the 3′ ends of transcripts to better understand regulatory mechanisms conferred by the 3′UTR and their roles in pathological conditions.

A recent study demonstrated that α-SYN transcripts have at least five different lengths of 3′UTR ranged from 290 to 2520 nucleotides [nt] and there are correlations between lengths of α-SYN 3′UTR and PD [15, 16]. Some of the single nucleotide polymorphisms (SNPs) that are located in the 3′UTR of α-SYN have been shown to be associated with sporadic PD [17, 18]. Together, the 3′UTR of α-SYN plays important roles in regulating α-SYN expression and eventually PD pathogenesis.

Recent accomplishment of the ENCODE (Encyclopedia of DNA Elements) provides comprehensive information on tissue-specific gene regulations. Data suggest that the last exon of *SNCA* might be much longer than the annotated length, generating α-SYN mRNA containing the extended 3′UTR. In this study, we sought to identify this extended α-SYN transcript in human postmortem brain tissues and various human neuronal cell lines and its role in translational regulation of α-SYN.

## Methods

### Post-mortem human brain samples

The use of post-mortem brain tissue was approved by the University of Central Florida Institutional Review Board. In the present study, 8 post-mortem brain samples without any neurodegenerative disease were used. The *substantia nigra* (SN) region containing brain tissues were obtained from the NIH Neurobiobank consortium. Ages ranged from 54 to 89 years and the post-mortem interval (PMI) varied from 10 to 30.25 h.

### Cell culture

#### iPSCs (induced pluripotent stem cells)

Four iPSC lines were generated from the skin fibroblast of control (SC1014, SC1015), and sporadic PD (ND35302, ND35322) obtained from Coriell Institute for Medical Research (Additional file 1: Table S1). Each iPSC lines had 2~ 5 clones from different batches of reprogramming. Using CytoTune® iPS 2.0 Sendai reprogramming protocol (Thermo Scientific), we reprogrammed fibroblasts into transgene-free iPSC. Fibroblasts were followed reprogrammed into transgene-free iPSC as we described previously [19]. The iPSC were cultured on cell cycle arrested mouse embryonic fibroblasts (MEF) feeder cells in human embryonic stem cell media containing DMEM/F12, 20% knockout serum replacement (KSR), basic fibroblast growth factor (bFGF, 4 ng/ml), glutamine (2 mM), non-essential amino acids (NEAA, 0.1 mM) and β-mercaptoethanol (0.1 mM). The iPSC lines were differentiated into dopaminergic neurons following our previous protocol [19].

#### ReNcell VM (Human ventral mesencephalic neuronal progenitor cells)

Cells were cultured on laminin-coated (20 μg/ml) dishes in maintenance medium containing DMEM/F-12 with B27 supplement, glutamax, heparin (10 U/ml), gentamicin (50 μg/ml), bFGF (20 ng/ml) and epidermal growth factor (EGF, 20 ng/ml).

#### SH-SY5Y cells

Cells were grown in DMEM/F12 medium containing 10% fetal bovine serum (FBS), penicillin (100 U/ml) and streptomycin (50 μg/ml).

### RT-PCR

Total RNAs were extracted from SN tissues using TRIzol and then they were treated with DNaseI (DNA-*free*™ Kit, Ambion) to remove contaminating genomic DNA. DNA-free RNAs were converted to cDNA using the amfiRivert cDNA Synthesis Kit (GenDepot) according to the manufacturer’s protocol. For negative RT reaction, nuclease-free water was added instead of RT enzyme. Synthesized cDNA samples were subject to PCR amplification of α-SYN using different sets of primers: F1 + R1, F2 + R2, F3 + R3, and β-actin. The following are primer sequence information: Human α-SYN Forward (F) 1; 5′-GTGGCTGCTGCTGAGAAAAC, Reverse (R) 1; 5′-CACCACTGCTCCTCCAACAT, F2; 5′-CTCCCGAGACATTCACCTGC, R2; 5′-TTTTGGTAAAGCCGACCGTG, F3; 5′-ACAGAAGCTATGAGTAACATGAGG, R3; 5′-TACACTCACTCACAACACTCAA, and β-actin F; 5′-GGAGTCCTGTGGCATCCACG, R; 5′-CTAGAAGCATTTGCGGTGGA.

Total RNAs from neuronal cell lines (ReNcell VM and SH-SY5 cells) were similarly extracted described above and RNAs from iPSCs derived dopaminergic neurons were extracted using the RNeasy Plus Mini Kit (Qiagen). Total RNAs from LUHMES cells were kindly given by Dr. Coetzee (Van Andel Research Institute). Received RNAs were treated with DNaseI and cDNA was generatedas above.

### 3′-Rapid Amplification of cDNA Ends (3′-RACE)

3 μg of total RNA were used for 3′-RACE reaction. First strand cDNA was synthesized using SuperScript™ II Reverse Transcriptase (ThermoFisher Scientific) with a QT primer containing a 17 nucleotide oligo-(dT) sequence at the 3′ end followed by a 35 nucleotide sequence. Then, first round amplification for α-SYN ends was done using α-SYN 3′RACE F1 and Q2 primer set. The first round product was diluted to 1:20 in a Tris-EDTA solution and used for the second round amplification using α-SYN 3′RACE F2 and Q1 primer set. Third round amplification was done as described above using a α-SYN 3′RACE F3 and Q1 primer set. Final PCR product was confirmed using gel electrophoresis (Fig. 3a) and sent out for sequencing analysis. The followings are primer sequence information: QT; 5′–CCAGTGAGCAGAGTGACGAGGACTCGAGCTCAAGCTTTTTTTTTTTTTTTTT, Q2; 5′–CCAGTGAGCAGAGTGACG, Q1; 5′–GAGGACTCGAGCTCAAGC, α-SYN 3′RACE F1; 5′-ACCAGAAAGGTCAAGCCATGATAAGAAGCTT, α-SYN 3′RACE F2; 5′-GTCTGTGAATCACACTAGCAAATTATCAAACCT, α-SYN 3′RACE F3; 5′-CAGAAGCTATGAGTAACATGAGGACTC.

Newly found sequence, the extended α-SYN 3′UTR, was reported to DDBJ/ENA/GenBank Databases. Nucleotide sequence data reported are available in the Third Party Annotation (TPA) Section of the DDBJ/ENA/GenBank databases under the accession number TPA: BK010481.

### Luciferase reporter constructs

To generate luciferase constructs carrying the annotated (2.5 kb) or extended (3.8 kb) α-SYN 3′UTR, 2.5 kb or 3.8 kb of human α-SYN 3′UTR with terminal MluI and PmeI restriction sites were PCR-amplified from genomic DNA using a Q5 high-fidelity DNA polymerase (NEB). Then enzyme digested PCR products were inserted into a pMIR-reporter luciferase vector (Ambion). Constructs were confirmed by sequencing before using.

### Luciferase assay

For the luciferase assay, SH-SY5Y cells were co-transfected with firefly luciferase constructs containing either 2.5 or 3.8 kb α-SYN 3′UTR along with Renilla luciferase in 24-well plates using jetPRIME (Polyplus Transfection). Cells were collected after 36 h post-transfection and dual luciferase assay was performed according to the manufacture’s protocol (Promega). Relative luciferase activity was calculated by normalizing activity obtained for firefly to Renilla. Experiment was repeated three independent times.

### Western blot

Protein concentrations were determined by the BCA assay (Pierce). Equal amounts of protein were electrophoresed on 8–16% gradient SDS-PAGE gels (Life technologies), transferred onto nitrocellulose membrane, and blocked with TBST (150 mM NaCl, 10 mM Tris-HCl pH 7.4, 0.05% Tween 20) containing 5% skim milk for 30 min. Primary antibodies were incubated at 4 °C overnight followed by HRP-conjugated mouse or rabbit secondary antibodies (1:5000, GE Healthcare) for 1 h at room temperature. Chemiluminescence (Thermo Scientific) was utilized to visualize the immunoblot signals. The following primary antibodies were used in this study: anti-α-SYN antibody (1:2000, 610787, BD Biosciences), anti-TH antibody (1:2000, NB300–19, Novus Biologicals) and HRP-conjugated anti β-actin antibody (1:50,000, A3854, Sigma).

### Bioinformatic analysis

RBPmap (Version 1.1) was used to predict the RBP binding sites in the extended α-SYN 3′UTR. Sequence of the extended 3′UTR was screened for Human/Mouse RBPs binding motifs with high stringency levels (*P*-value < 0.001), yielding a total 74 RBPs. They were ranked by expression levels in the brain compared to other tissues based on HPA RNA-seq normal tissues data [20]. Some of them have been reported by their roles in the brain. Together with results of their expression levels and their roles in the brain, a total of 13 RBPs were selected and listed in Table 1.

Prediction of miRNAs targeting the extended α-SYN 3′UTR sequence was done through miRDB with custom prediction (http://www.mirdb.org). miRNAs with the highest target prediction scores (> 70) obtained by MirTarget algorithm were depicted in Fig. 3b. and their expression levels in human brain tissue were checked using the human miRNA expression database (miRmine) [21].

PD implicated SNPs in extended 3′UTR were examined in regulome SNP database (http://regulomedb.org), as well as in the UCSC genome. Eight SNPs were found and four of them (rs7675290, rs8180214, rs8180209, rs17016071) were shown to be in tight linkage disequilibrium (r^2^ ≥ 0.95; in several populations) with a previously reported lead SNP (rs11931074) described in a large Japanese cohort using genome wide association studies with PD (http://regulomedb.org/GWAS/rs11931074_r2thr0.8_all.html) [22].

### Statistical analysis

Statistical analysis was performed with GraphPad Prism v.7.04 (GraphPad Software). Data are presented as mean ± S.E.M of each experimental condition. Two-tailed unpaired t test was performed in each experimental condition. To determine the correlation between extended α-SYN 3′UTR with α-SYN protein expression, two-tailed Pearson’s correlation was used for the groups followed by linear regression analysis. Values of *p* < 0.05 were considered significant.

## Results

**Fig. 1:**
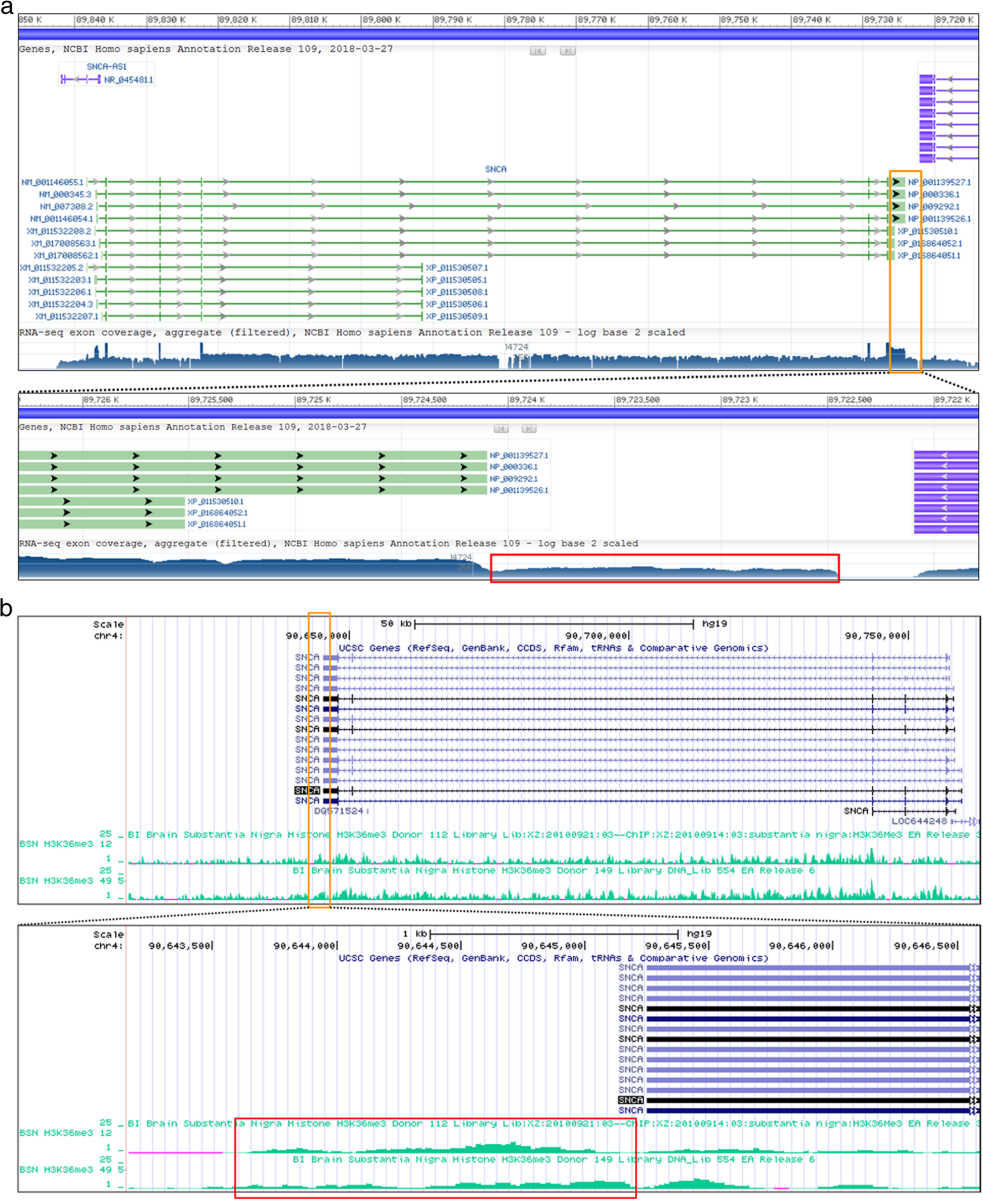
RNA-seq data of α-SYN transcripts and H3K36me3 histone distribution of the *SNCA* gene. **a** RNA-seq coverage data of *SNCA* in NCBI *Homo sapiens* Annotation (GRCh38.p12 assembly) is shown. Data near the last exon of *SNCA* (Orange box) is shown in the lower panel. Red rectangular box indicates the predicted extension of α-SYN 3′UTR. **b** The H3K36me3 distribution data of *SNCA* from two adult postmortem SN tissues collected by the NIH Roadmap Epigenomics Mapping Consortium [28, 29] is shown. The UCSC genome browser image (GRCh37/hg19 assembly) was obtained according to the instruction of the data table page. Data near the last exon of *SNCA* (Orange box) is shown in the lower panel. Red rectangular box indicates continuous H3K36me3 coverage after the annotated exon of *SNCA*. Note that transcriptional direction is from right to left

RNA-sequencing (RNA-seq) alignments on a genetic locus around the last exon of *SNCA* in NCBI *Homo sapiens* Annotation Release 109 shows the existence of RNA-seq reads on the region after the end of annotated last exon (Fig. 1a). In this same region of *SNCA*, peaks of trimethylated histone H3 at lysine 36 (H3K36me3) are also enriched (Fig. 1b). H3K36me3 has been known to indicate actively transcribed regions of the gene body [23, 24]. Therefore, together with RNA-seq coverage data and the continuous H3K36me3 on a genomic locus beyond the annotated last exon of *SNCA* implies existence of the extended 3′UTRs of α-SYN. Based on this analysis (Fig. 1a and b), we roughly estimated the length of the extended α-SYN 3′UTR to be about 1500 nt.

**Fig. 2:**
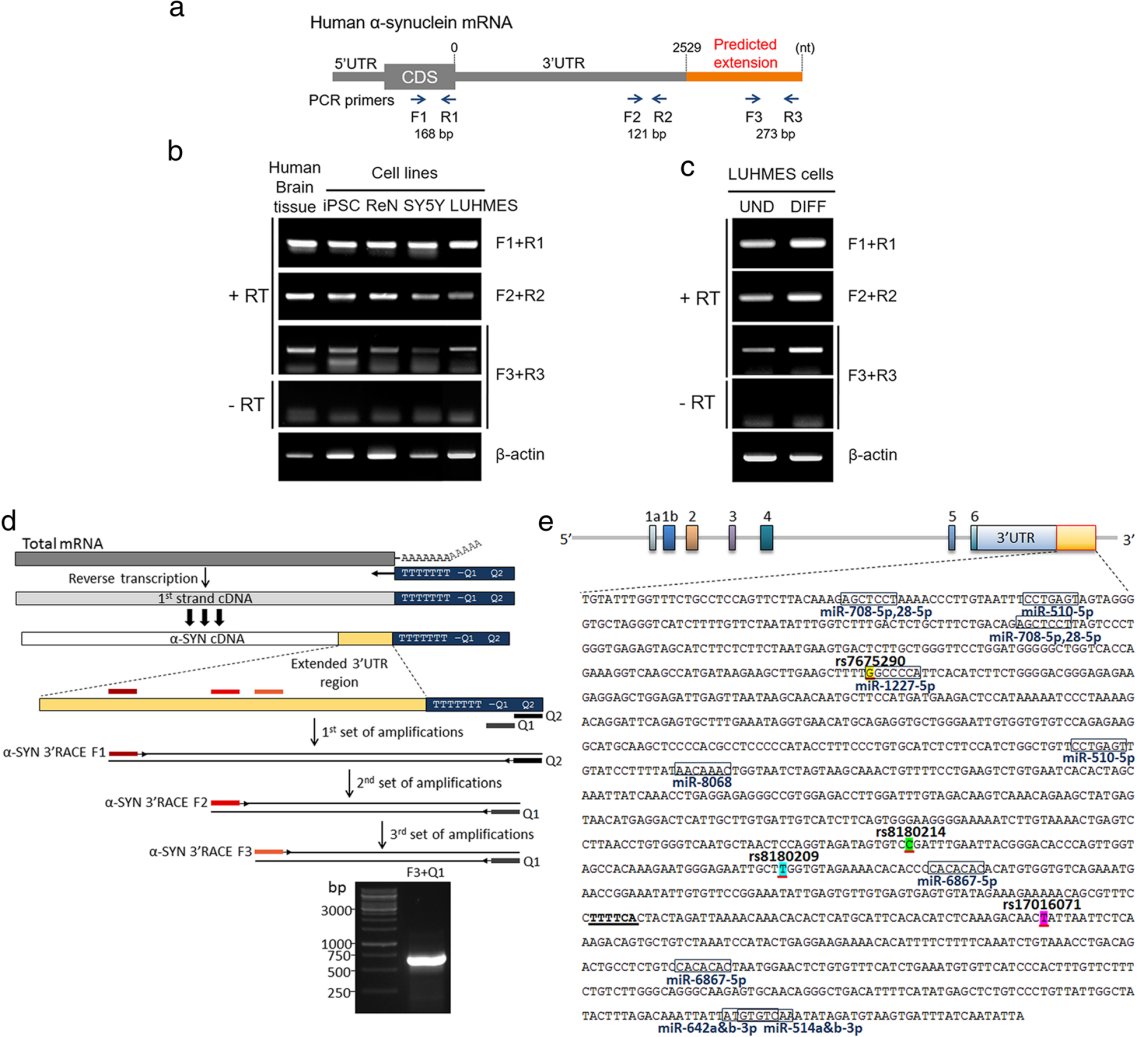
PCR amplification and the sequence of the extended 3′UTR of α-SYN mRNA and the potential regulatory miRNAs and SNPs. **a** The schematic structure of α-SYN mRNA including the predicted extension of 3′UTR. Three different primers sets for PCR amplification are shown. CDS; coding DNA sequence. **b** RT-PCR for the extended 3′UTR in the SN tissue of postmortem brains (one representative sample is shown here out of 8 brain samples) and other human neuronal cell lines: iPSC, dopaminergic neurons (DIV 60) differentiated from induced pluripotent stem cells; ReN, human ventral mesencephalic neuronal progenitor cells; SY5Y, human neuroblastoma cells; LUHMES, immortalized human dopaminergic neuronal precursor cells. “+” or “-” RT; with or without RT reaction. **c** Expression of the extended 3′UTR in undifferentiated (UND) and differentiated (DIFF) LUHMES cells. **d** Schematic overview of the 3′-RACE procedure. Three serial amplification steps using three forward and two reverse primers were performed to amplify the terminal region of extended α-SYN 3′UTR. **e** Schematics of *SNCA* gene structure including the newly identified end of the last exon with yellow box. The sequence of the extended 3′UTR with marks for binding sites of miRNAs and SNPs are shown. miRNAs with the high target prediction scores (> 70) are marked. The four SNPs highlighted in the extended 3′UTR, are in significant linkage disequilibrium (r^2^ ≥ 0.95) with the PD-implicated SNP (rs11931074) in various populations. The distances of these indicated SNPs from the lead SNP (rs11931074) are as follows: rs7675290 is 5488 bp; rs8180214 is 4993 bp; rs8180209 is 4939 bp and rs17016071 is 4766 bp

In order to identify this extended α-SYN transcript, we designed three different sets of reverse transcriptase-PCR (RT-PCR) primers targeting the protein coding region, the distal region of the known 3′UTR, and the predicted extended 3′UTR; F1 + R1, F2 + R2, and F3 + R3, respectively (Fig. 2a). RT-PCR was performed on the SN tissue of postmortem brains, human iPSCs-derived dopaminergic neurons, ReNcell VM (human ventral mesencephalic neuronal progenitor cells), SH-SY5Y cells (human neuroblastoma cells), and LUHMES cells (immortalized human dopaminergic neuronal precursor cells). Regardless of the types of cell lines or brain tissue, the extended 3′UTR was successfully amplified (Fig. 2b). To exclude the possibility of genomic DNA amplification, the same sets of RNA samples without RT reactions (shown as “- RT”) were included, confirming no genomic DNA contamination. Moreover, we compared the expression levels of the extended 3′UTR before and after differentiation of LUHMES cells. Interestingly, α-SYN transcript containing the extended 3′UTR was proportionally increased as LUHMES cells were differentiated (Fig. 2c).

Next, to find the last sequence of the extended α-SYN 3′UTR region, 3′-Rapid Amplification of cDNA Ends (3′-RACE) was performed (Fig. 2d). With DNA sequencing, we confirmed that the extended 3′UTR contains an additional 1246 nt after the longest known annotated α-SYN 3′UTR (2529 nt) (Fig. 2e). Our findings extend the end of the last exon of *SNCA* by 1246 bp that can generate human α-SYN mRNA having the maximum 3775 nt-length 3′UTR.

**Fig. 3:**
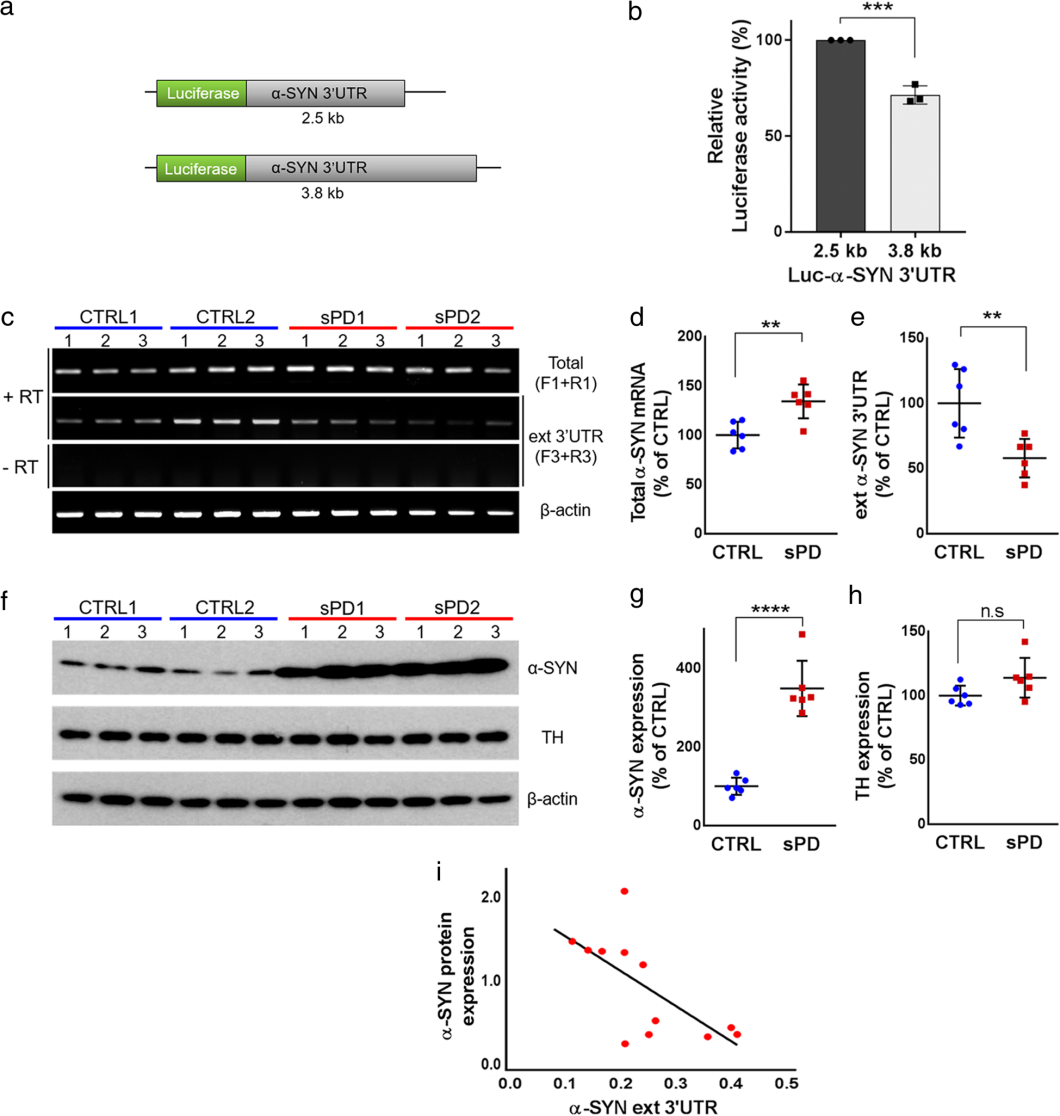
The effect of the extended α-SYN 3′UTR on α-SYN translation and their level changes in iPSC-derived dopaminergic neurons. **a** Firefly luciferase reporter constructs containing the annotated (2.5 kb) or extended (3.8 kb) form of α-SYN 3′UTR. **b** Luciferase activity from SH-SY5Y cells co-transfected with firefly luciferase containing either 2.5 or 3.8 kb α-SYN 3′UTR and Renilla luciferase. The firefly luciferase values were normalized to Renilla luciferase activity. **c** RT-PCR for total α-SYN transcripts, the extended α-SYN 3′UTR and β-actin from iPSC-derived dopaminergic neurons (DIV 60). RNA samples from total 12 iPSC lines; three iPSC clones from each patient (two control; CTRL1 and 2, two sporadic PD; sPD1 and 2), were used. β-actin was used as an internal control. “+” or “-­” RT; with or without RT reaction. **d** Quantitative analysis of total α-SYN mRNA expression after normalization by β-actin. **e** Quantitative analysis of the extended α-SYN 3′UTR expression after normalization by β-actin. **f** Western blotting for α-SYN, tyrosine hydroxylase (TH), and β-actin from iPSC-derived dopaminergic neurons (DIV 60). β-actin was used as an internal control. **g** Quantitative analysis of α-SYN protein expression after normalization by β-actin. **h** Quantitative analysis of TH protein expression after normalization by β-actin. **i** Reverse correlation between the extended α-SYN 3′UTR and α-SYN protein levels. The Pearson’s correlation coefficient = − 0.6688. Error bars denote mean ± S.E.M. n.s (not significant), **P < 0.01, ***P < 0.001, ****P < 0.0001 by unpaired two-tailed t test in **d**, **e**, **g**, **h**

To explore whether the extended α-SYN 3′UTR affects translation of α-SYN, we generated luciferase reporter constructs carrying the annotated (2.5 kb) or extended (3.8 kb) α-SYN 3′UTR (Fig. 3a). Firefly luciferase constructs containing either 2.5 or 3.8 kb α-SYN 3′UTR along with Renilla luciferase were transfected into SH-SY5Y cells. Luciferase activity was significantly lower in cells transfected with the extended α-SYN 3′UTR compared to the 2.5 kb α-SYN 3′UTR (Fig. 3b), suggesting that additional *cis*-elements in the extended α-SYN 3′UTR negatively regulate translation of α-SYN. Next, we investigated the expression levels of extended α-SYN 3′UTR from iPSC-derived dopaminergic neurons from sporadic PD patients and control subjects. Surprisingly, level of the extended α-SYN 3′UTR from sporadic PD iPSC-derived dopaminergic neurons (sPD) was significantly lower than one from control dopaminergic neurons (CTRL) (Fig. 3c and e), even though total α-SYN mRNA levels were higher in sPD (Fig. 3c and d). On the other hand, α-SYN protein levels were significantly increased in sPD without changing tyrosine hydroxylase levels (Fig. 3f, g and h). The strong negative correlation between the expression of the extended α-SYN 3′UTR and α-SYN protein levels was found in these iPSC-derived dopaminergic neurons (Fig. 3i).

The length of the 3′UTR affects its translation, localization, and stability through providing binding sites for RBPs and miRNAs [7]. Altered expression of RBPs may affect the 3′UTR length. Therefore, it is worth to explore the potential regulatory *cis*- elements and cognate *trans*-factors, RBPs, present in the extended 3′UTR. Predicted RBPs using RBPmap [25] were ranked according to their brain-specific expression levels and known roles in the brain. The 13 highest-ranked RBPs which are feasible in regulating α-SYN in the brain are listed in Table 1. Next, we investigated the number of miRNA binding sites in this region using the MirTarget algorithm (miRDB) [26, 27]. Ten miRNAs showing the highest target prediction score (> 70) are depicted with their binding sites (Fig. 2e). Among them, has-miR-708-5p and has-miR-28-5p have high expression in human brain tissues [21]. We also have looked for SNPs in this extended region from the SNP database (RegulomeDB and UCSC genome) in search of PD association. We found four SNPs: rs7675290, rs8180214, rs8180209, and rs17016071. These four SNPs are in strong linkage disequilibrium (r^2^ ≥ 0.95) with a downstream disease implicated SNP (rs11931074) that is strongly associated with PD in all HapMap 2 populations as found in the genome-wide association study by Satake et al., 2009 (Fig. 2e) [22].

**Table 1:** Potential RNA binding proteins for the extended α-SYN 3′UTR. RBPs highly expressed in the human brain with their binding motifs in the extended α-SYN 3′UTR

Protein name	Full protein name	Target RNA motifs
CELF4;BRUNOL4	CUGBP Elav-like family member 4	kgugukk
CELF5;BRUNOL5	CUGBP Elav-like family member 5	ugugukk
CELF6;BRUNOL6	CUGBP Elav-like family member 6	ugugdkg
CNOT4	CCR4-NOT transcription complex subunit 4	gacaga
FXR1	FMR1 autosomal homolog 1	aygacr
HuR;ELAVL1	ELAV like RNA binding protein 1	uukruuu
MATR3	matrin 3	maucuur
MSI1	musashi RNA binding protein 1	uaguwrg
NOVA1	NOVA alternative splicing regulator 1	ycay
PUM2	pumilio RNA binding family member 2	uguanaua
QKI	QKI, KH domain containing RNA binding	acuaay
RBFOX1	RNA binding protein, fox-1 homolog 1	wgcaugm
RBM28	RNA binding motif protein 28	gwguagd

## Discussion

The current reference sequence length of the longest human α-SYN 3′UTR is 2529 nt. In the current study, we identified α-SYN transcript having a much longer 3′UTR—an additional 1246 nt, in fact— in postmortem human brain samples and iPSCs-derived dopaminergic neurons, as well as various human neuronal cell lines, further extending the last exon of *SNCA*.

Recent studies have demonstrated that mRNAs containing the longer 3′UTRs, on average, were exclusively observed in the nervous system when compared to other tissues, suggesting neuron-specific functions of the extended form of 3′UTR [12–14]. The longer extended form of α-SYN 3′UTR identified in this study confers an additional intricate regulation that might be important for specific neuronal functions or pathological conditions. In fact, we found that the longer extended α-SYN 3′UTR is expressed more in differentiated LUHMES cells when compared to undifferentiated conditions. The extended form of α-SYN 3′UTR, however, was decreased in sporadic PD iPSC-derived dopaminergic neurons in contrast to increased α-SYN protein levels. These suggest that aberrant expression of the extended α-SYN 3′UTR may influence PD pathogenesis.

Changes in RBPs may affect the production of 3′UTR with different lengths. We found 13 potential RBPs that can bind to the extended 3′UTR of α-SYN based on high expression levels in the brain. In addition, 10 miRNAs were predicted to bind to this region. Further studies are necessary to determine the mechanisms underlying regulation of the extended α-SYN 3′UTR, which might bring new therapeutic targets for human diseases especially in PD.

Finally, several annotated SNPs were found in the newly identified 3′UTR region. Four SNPs, although not evaluated in PD, are shown in significant linkage disequilibrium (r^2^ ≥ 0.95) with another lead SNP (rs11931074) downstream of the gene. This SNP was significantly associated with PD in the Japanese population in a large GWAS study [22]. Moreover, these four SNPs were originally described as being located in the “intergenic region” on chromosome 4. With our discovery of the extended 3′UTR of *SNCA*, these variants will now be included within the *SNCA* 3′UTR region.

In summary, the present study identified the longer extended form of human α-SYN 3′UTR and its negative correlation with α-SYN translation. These findings would bring forth a better understanding not only how α-SYN expression is regulated but also how the length of α-SYN 3′UTR is involved in PD pathogenesis.

## Additional file

Additional file 1: **Table S1.** The information of six iPS cell lines from Coriell Institute for Medical Research. (DOCX 16 kb)

## Abbreviations

3′-RACE: 3′-Rapid Amplification of cDNA Ends
3′UTRs: 3′ untranslated regions
H3K36me3: Trimethylated histone H3 at lysine 36
iPSC: Induced pluripotent stem cell
LBs: Lewy bodies
LNs: Lewy neurites
miRNAs: MicroRNAs
mRNAs: Messenger RNAs
nt: Nucleotides
PD: Parkinson’s disease
RBPs: RNA binding proteins
SN: Substantia nigra
SNPs: Single nucleotide polymorphisms
TH: Tyrosine hydroxylase
TPA: Third Party Annotation
α-SYN: Alpha-synuclein
