# Supplementary material for: A novel extended form of alpha-synuclein 3′UTR in the human brain
Source: Mol Brain. 2018 May 25;11:29. doi: 10.1186/s13041-018-0371-x (PMC5970512; doi:10.1186/s13041-018-0371-x)
Supplement: Supplementary file 1 — Table S1. The information of six iPS cell lines from Coriell Institute for Medical Research. (DOCX 16 kb) [file 13041_2018_371_MOESM1_ESM.docx]

**Table S1.** The information of six iPS cell lines from Coriell Institute for Medical Research

| iPS cell clone ID | Coriell ID (Fibroblast) | Genotype | Diagnosis |
| --- | --- | --- | --- |
| C1 | SC1014 | wt/wt | Health condition |
| C2 | SC1015 | wt/wt | Health condition |
| sPD1 | ND35322 | wt/wt | PD |
| sPD2 | ND35302 | wt/wt | PD |
